# Application of Space-Time Cube Analysis to Brewing Water Resources: A Complementary Decision-Support Tool for Breweries

**DOI:** 10.3390/foods15112021

**Published:** 2026-06-04

**Authors:** Eugenia Iturritxa, Annie E. Hill, María-Jesús Torija

**Affiliations:** 1 Universitat Rovira i Virgili, Departament de Bioquímica i Biotecnologia, Grup de Recerca Biotecnologia Enològica, Facultat d’Enologia, c/Marcel⋅lí Domingo, 1, 43007 Tarragona, Spain; 2 NEIKER, Basque Institute for Agricultural Research and Development Department of Forest Science, Neiker-BRTA, Instituto Vasco de Investigación y Desarrollo Agrario, Granja Modelo s/n, Antigua Carretera Nacional 1, Km. 355, 01192 Arkaute, Spain; 3 International Centre for Brewing & Distilling, Heriot-Watt University, Edinburgh EH14 4AS, UK; a.hill@hw.ac.uk

**Keywords:** brewing process, water quality, space-time cube tools, Basque Country

## Abstract

Brewing water must meet potability standards, as its chemical composition directly affects beer quality and process consistency. Understanding spatial and temporal variability in water composition is essential for quality control and risk management in brewing. This study evaluated the spatiotemporal variation of six key brewing parameters (pH, calcium, magnesium, sodium, chloride, and sulphate) in the Basque Country (Spain) from 2019 to 2023. A comprehensive dataset of drinking water analyses from multiple municipalities was assessed using spatiotemporal analysis tools to identify trends, variability patterns, and potential deviations from recommended brewing water ranges. Water composition showed notable spatial variability, while temporal fluctuations were generally limited. Most parameters remained within recommended ranges for brewing. However, isolated exceedances were identified for sulphate (two municipalities) and sodium (one municipality), representing less than 0.06% of the total records. These deviations may affect flavor profiles and process stability if not properly managed. The findings highlight the importance of continuous monitoring of source water to ensure consistent brewing quality, despite limitations associated with heterogeneous monitoring practices and multi-year data aggregation. The proposed spatiotemporal approach supports risk-based decision-making for brewery location, contributing to improved quality control in the brewing industry.

## 1. Introduction

Water is a major component of beer, and its characteristics determine the efficiency and flavor of the extracted wort, directly contributing to flavor and impacting the perceived bitterness and hop utilization in the finished beer. Additionally, ions in water are critical to the mashing process. Therefore, maintaining consistent water quality is essential for ensuring product quality and process control in brewing.

Water quality and components may vary depending on the environmental conditions, levels of human activity, and water sources. For instance, precipitation typically has a lower pH and mineralization than surface and groundwater. Surface water (from rivers, lakes, and reservoirs) may contain higher levels of organic contaminants depending on location and anthropogenic activities, whereas groundwater, although generally lower in organic contamination, may be affected by industrial and agricultural pollutants [1]. Furthermore, geological formations influence the types and concentration of dissolved minerals [2]. These variations highlight the importance of monitoring source water to ensure consistent quality and suitability for food production processes such as brewing. Regular assessment of drinking water is essential to comply with legal potability requirements and ensure consumer safety. Distribution systems, divided into supply zones, are typically monitored at multiple sampling points, with sampling frequency and parameters defined by regulatory authorities and public health agencies. Water quality assessment is based on the statistical analysis of the physical, chemical, and biological results [3]. In this context, systematic monitoring also supports process control and risk management in food-related industries.

Public health centers, along with control and surveillance units, form the Basque Country’s Public Drinking Water Control and Surveillance Network, operating under the framework established by European Directive (EU) 2020/2184 [4]. This system ensures compliance with drinking water standards while providing valuable data for evaluating spatial and temporal variability in water quality.

Key parameters influencing the brewing process include pH, calcium, magnesium, sodium, chloride, and sulphate. The pH is typically between 5.2 and 9.5 at 20 °C [1]. The pH provides information on the water origin and treatment, and influences mash conditions. Calcium and magnesium interact with malt phosphates, affecting the mash pH. Calcium facilitates flocculation by affecting cell–cell interactions and promoting protein coagulation [5]. Magnesium, generally involved in enzymatic yeast metabolism and alcohol production, can lend a sour and bitter aftertaste at concentrations >50 ppm [5]. Chloride ions enhance the malty aspects of beer, sweetness, and mouthfeel perception, with levels above 250 ppm producing a doughy, salty taste, apart from affecting the yeast health. Conversely, sulphate ions tend to accentuate the flavor and bitterness [6]. Chloride concentrations above 200 ppm result in a full malt flavor, while sulphate (>200 ppm) are optimal for hoppy beers, such as India Pale Ale [7].

A sulphate/chloride ratio of approximately 1:1 (or 1.0) is considered balanced because water does not enhance malt or hop flavor. Ratios lower than 1 tend to increase malt characteristics, while ratios > 1 enhance the hop characteristics. Ratios of 1:2 are suggested for bitter styles, 2:1–2:3 for mild ales and stouts, and 3:1 for better-tasting porters [5,8].

Sodium contributes to beer’s perceived taste by enhancing its sweetness. Levels of 75–150 ppm provide rounded smoothness and accentuated sweetness, which is more pleasant when combined with chloride ions than with sulphate ions. Sodium creates unpleasant harshness with sulphate [9].

Given the variability in municipal water characteristics due to environmental, seasonal, and operational factors, advanced analytical approaches are needed to better understand their behavior over time and space.

Several aspects of brewing water variability in the Basque Country remain poorly understood, particularly the true spatial and temporal heterogeneity of hydrochemical composition at the regional scale. Traditional drinking-water monitoring programs are primarily designed to ensure regulatory compliance and public health safety rather than to characterise hydrochemical diversity relevant to brewing applications. Consequently, monitoring intensity is often concentrated in the largest supply systems, while smaller or local water sources are sampled less frequently and with lower analytical coverage. In addition, not all chemical parameters are measured consistently across municipalities or time periods, resulting in uneven datasets and limiting comparability among locations. This monitoring structure may mask local variability, reduce the detection of hydrochemical diversity, and promote an apparent homogenisation of regional water profiles. As a result, conventional analyses are often insufficient to identify medium- and long-term spatial patterns, emerging temporal shifts, or niche hydrochemical conditions relevant to beer style selection and mineral adjustment strategies.

Spatiotemporal analysis allows the identification of patterns, trends, and anomalies in water quality data, supporting improved monitoring and decision-making. The space-time cube model, originally proposed by Hägerstrand [10], enables the simultaneous analysis of spatial and temporal dimensions by aggregating data into structured intervals. The space-time cube aggregates events into intervals, accurately representing spatiotemporal data in time-based groupings such as new, consecutive, persistent, oscillating, and sporadic hot or cold spots [11]. Spatiotemporal cube tools have been crucial for understanding different multidisciplinary events such as the spread of COVID-19 [12], urban mobility patterns [13], human conflicts [14], and ecological studies [15,16].

In this context, the present study aims to monitor the spatiotemporal evolution of six key water chemistry variables relevant to the brewing process across the Basque Country between 2019 and 2023 using ArcGIS Pro 3.2.2 Space-Time Cube analytical tools. By integrating medium- to long-term spatiotemporal trend analysis with brewing-oriented hydrochemical interpretation, this work seeks to provide an additional regional-scale decision-support tool complementary to conventional brewery-specific water analyses. The study further aims to identify spatial patterns, emerging temporal changes, and regional hydrochemical variability associated with brewing suitability and mineral adjustment strategies, while establishing a methodological framework that may support future integrated studies incorporating detailed geological, hydrogeological, and environmental datasets for quantitative assessment of hydrochemical drivers.

## 2. Materials and Methods

### 2.1. Data Source

The data used in this study were obtained from drinking water analyses in the Basque Country, Spain (available from the Basque Government, Basque Country, Spain). The databases were downloaded and processed to determine the spatiotemporal variation by municipality and year quartiles. The spatiotemporal variation of these parameters was monitored for five years (2019–2023).

### 2.2. Data Preprocessing

The initial raw data were downloaded and reviewed to select municipalities that met the requirements of the process. Hydro-chemical data were preprocessed to ensure temporal consistency and analytical reliability. Decimal separators and measurement precision were standardised to remove spurious outliers caused by formatting errors. Time series with missing observations were excluded. The data were then aggregated into five-year quartile intervals and mean values to capture long-term variability while reducing analytical noise. To minimise information loss from incomplete multivariate coverage across monitoring locations, separate space-time cubes were generated for each chemical variable (major ions and pH), preventing bias from mismatched missing-data patterns and maximising spatial and temporal data retention. The data were analysed and grouped by municipality and quartile from 2019 to 2023 before creating space-time cubes for each variable. The quartile distribution allowed the visualisation of changes in water sources owing to the possible seasonal impact in each municipality [17]. Variables were analysed separately to maintain the database (data for some variables were unavailable for the same municipalities), and to comply with the implementation requirements of the space-time cube tools. Data availability varied among hydrochemical parameters, with spatial coverage ranging from 27.8% to 96.4% of the 252 municipalities analysed. Major ions exhibited moderate and heterogeneous coverage (SO_4_^2−^: 35.3%, Na^+^: 32.5%, Mg^2+^: 27.8%, Ca^2+^: 35.7%, Cl^−^: 32.9%), whereas pH data were available for the vast majority of municipalities (96.4%). This uneven spatial and temporal availability motivated the construction and analysis of separate space-time cubes for each variable, ensuring that each parameter was evaluated using its maximum available information and avoiding biases associated with incomplete data overlap. This approach improves the robustness and interpretability of the identified spatiotemporal patterns.

### 2.3. Creation and Visualisation of the Space-Time Cubes

This study adopted the space-time cube algorithm in ArcGIS Pro (Esri, CA, USA) [18]. A space-time cube for each variable was created from defined locations and municipalities, allowing visualisation of spatiotemporal data through time-series analysis, spatial and temporal pattern analysis, and two-dimensional (2D) and three-dimensional (3D) visualisation techniques. More than 60 locations and at least 10, but fewer than 30 time-steps were required. The parameters were selected based on the availability of data and their strong influence on beer quality and style. Six parameters were included in this study: calcium, sodium, magnesium, sulphate, chloride, and pH.

The netCDF data cube for each variable was used to calculate the initial summary statistics and trends. In addition, space-time cubes were used as inputs in the space-time pattern analysis (Time-Series Clustering and The Emerging Hot Spot Analysis tools) and time-series forecasting (forest-based forecast) toolsets.

### 2.4. Time-Series Clustering

This tool classifies the most similar locations in a space-time cube and defines clusters with similar time-series characteristics. The simplest option is to cluster time series with similar values over time. This option measures the similarity of the time series using the Euclidean distance between their values. This value was calculated as the square root of the sum of the squared differences in values over time. The locations of the space-time cubes were clustered using the k-means algorithm. This algorithm can be applied without calculating the difference between pairs of locations. It began by randomly selecting locations to represent each cluster. The initial clusters were then generated by assigning all remaining locations to the cluster whose representative was most similar to the location. A new representative value for each cluster was calculated by averaging the time series within each cluster. For the value, the new representative was the average of each time step of each time series in the cluster. The number of cluster parameters was left blank, and the tool evaluated the optimal number of clusters by attempting different values and identifying the one that yielded the most effective clustering [19,20].

The tool tests each value between two and ten clusters, repeating each value 10 times with random starting values in the clustering algorithms. A pseudo-F statistic was calculated by dividing the squared errors of the global medoid by those of the cluster medoids, correcting for the use of larger numbers of clusters. This can be interpreted as the ratio of the between-group to within-group similarities. Larger values of the pseudo-F statistic indicate that the time series is more similar to the representative time series of its cluster than to those of the entire dataset, indicating effective clustering [21,22].

Clustering effectiveness was measured using the Caliński–Harabasz pseudo-F-statistic [23], which is the ratio of the between-cluster variance to the within-cluster variance. Notably, the ratio reflects within-group similarities and between-group differences:

(1) $$\frac{\left(\frac{R^{2}}{n_{c}-1}\right)}{\left(\frac{1-R^{2}}{n-n_{c}}\right)}$$

Being R^2^:

(2) $$R^{2}=\frac{SST-SSE}{SST}$$

SST = sum of squares total, reflection of differences between clusters.

SSE = sum of squares error, reflection of differences within the cluster.

(3) $$SST=\sum_{i=1}^{n_{c}}\sum_{j=1}^{n_{i}}\sum_{k=1}^{n_{v}}{\left(V_{ij}^{k}-\bar{V^{k}}\right)}^{2}$$

(4) $$SSE=\sum_{i=1}^{n_{c}}\sum_{j=1}^{n_{i}}\sum_{k=1}^{n_{v}}{\left(V_{ij}^{k}-\bar{V_{i}^{k}}\right)}^{2}$$

n = number of features

n_i_ = number of features in cluster i

n_c_ = number of clusters

n_v_ = number of variables used to cluster features

$V_{\mathrm{i}j}^{k}$ = value of the kth variable of jth feature in the ith cluster

$\bar{V^{k}}$ = mean value of kth variable

$\bar{V_{i}^{k}}$ = mean value of kth variable in cluster i

This tool creates a new Output Feature Class with the following attributes for each feature in the Input Feature Class: Local Moran’s I index [24], z-score, pseudo *p*-value, and cluster type.

Clusters were defined as those showing similar values over time. The space-time NetCDF cubes created using the Create Space-Time cube from defined locations (municipalities) for each variable were taken as inputs. The tool generated a 2D feature class that shows each location in a cube, symbolised by its cluster classifications. The charts and tables show the representative time-series values for each cluster [21].

The Mann–Kendall trend test [19,20] was performed at each location using the data as an independent bin time series. The Mann–Kendall statistic is a rank correlation analysis of bin counts or values and their time sequences [25]. The bin value for the first time period was compared with that for the second time period. If the first was smaller than the second, the result was +1, while in the opposite case, the result was −1. If the two values were equal, the result was zero. The results for each pair of time periods were summed. The expected sum was zero, indicating no trend in the values over time. Based on the variance of the values in the bin time series, the number of ties, the number of time periods, and the observed sum were compared with the expected sum (zero) to determine whether the difference was statistically significant. The trend for each bin of the time series was recorded as a *z*-score and *p*-value. The sign associated with the z-score determines whether the trend depicts an increase in bin values (positive z-score) or a decrease in bin values (negative *z*-score).

### 2.5. The Emerging Hot Spot Analysis

The Emerging Hot Spot Analysis tool identified trends implemented for all variables. The tool takes a space-time NetCDF cube as input and uses the conceptualisation of spatial relationship values to calculate the Getis–Ord Gi* statistic, measuring local and global spatial autocorrelation for each bin, and uses the false discovery rate procedure to adjust the critical *p*-value thresholds.

The calculations for the Getis–Ord Gi* statistic are as follows:

(5) $$G_{i}^{*}=\frac{\sum_{j=1}^{n}w_{i,j}\;x_{j}-\bar{X\sum_{j-1}^{n}w_{i,j}}}{S\sqrt{\frac{\left[n\sum_{j=1}^{n}w_{i,j}^{2}-{\left(\sum_{j=1}^{n}w_{i,j}\right)}^{2}\right]}{n-1}}}$$

where x_j_ is the attribute value for feature j, w_ij_ is the spatial weight between feature i and j, and it is equal to the total number of features, and:

(6) $$\bar{X}=\frac{{\sum_{j=1}^{n}x}_{j}}{n}$$

(7) $$S=\sqrt{\frac{\sum_{j=1}^{n}x_{j}^{2}}{n}-{\left(\bar{X}\right)}^{2}}$$

The Getis–Ord statistics, known as Gi*, is a z-score used in spatial analysis to measure both local and global spatial autocorrelation. It is applied in Hot Spot Analysis to identify areas where features with high or low values are spatially clustered in a statistically significant manner [26].

Once the space-time Hot Spot Analysis was completed, each bin in the input netCDF cube had a corresponding *z*-score, *p*-value, and hotspot bin classification. Hot- and cold-spot trends were evaluated using the Mann–Kendall trend test [25] (Table 1). With the resultant trend *z*-score and *p*-value for each location with data and the hot spot z-score and *p*-value for each bin [27], the Emerging Hot Spot Analysis tool classified each study area location as described in https://pro.arcgis.com/en/pro-app/latest/tool-reference/space-time-pattern-mining/learnmoreemerging.htm (accessed on 18 January 2026) [28]. Additionally, Hotspot analysis enables two-dimensional or three-dimensional visualisation of the space-time cube, facilitating the visual identification of spatiotemporal data patterns.

### 2.6. Forest-Based Forecast

A forest-based forecast tool forecasted the future time slices of the space-time cubes of the variables. It was constructed by building a forest with time-series values at each location of the space-time cube, which was used to predict the next time slice. The forecasted value at the new time step was included in the forecast model, and the next time step was predicted. This recurrent process was extended to all future steps. This is an adaptation of the random forest algorithm developed by Breiman [29] and Cutler [30]. A single model trained from all locations was built, with outliers used to identify the locations and times that deviated significantly from the remaining time series [31].

This tool allows the construction of a single global forecast model that uses each location as the training data. The forest regression model was trained using time windows at each location of the space-time cube.

The forecast root-mean square error (RMSE) measures only how well the forest model fits the raw time-series values, which are equal to the square root of the average squared difference between the forest model and the values of the time series.

The equation for the Forecast RMSE is:

(8) $$Forecast\;RMSE=\sqrt{\frac{\sum_{t=1}^{T}{\left(c_{t}-r_{t}\right)}^{2}}{T}}$$

where T is the number of time-steps, ct is the value of the forest model, and rt is the raw value of the time series at time t.

The validation model was used to assess its ability to forecast future values for each time series. It was constructed by excluding some of the final time steps from each time series and fitting the forest model to the remaining data. This forest model was then used to forecast the values of the withheld data, and the forecasted values were compared with the hidden raw values. Four time-steps were withheld for validation, using the number of time-steps to be excluded as validation parameters (the number of time-steps excluded did not exceed 25% of the total). The accuracy of the forecasts was measured by calculating a Validation RMSE statistic, which is calculated as:

(9) $$Validation\;RMSE=\sqrt{\frac{\sum_{t=m+1}^{T}{\left(c_{t}-r_{t}\right)}^{2}}{m}}$$

where T is the number of time-steps, m is the number of time-steps withheld for validation, ct is the forecast value from the first T-m time-steps, and rt is the raw value of the time series withheld for validation at time t.

### 2.7. Multivariate Statistical Approach for Hydro-Chemical Guidance in Brewing

The combination of these analytical tools provides practical hydro-chemical guidance for beer style selection and the design of appropriate water adjustment strategies in brewing. Spatial analysis was conducted using municipal-level mean values of the chemical variables as an illustrative example, demonstrating the potential of the approach and its extrapolation to the full set of results derived from the ArcGIS Pro Space-Time Cube analytical toolkit.

Municipal drinking-water chemistry was characterised using mean concentrations of major ions (Ca^2+^, Mg^2+^, Na^+^, Cl^−^, SO_4_^2−^; mg·L^−1^) and pH (dimensionless). Municipal identifiers were harmonised across chemical, administrative, and geographic datasets. Municipalities with insufficient data coverage were excluded if they lacked numeric values for at least three of the six variables. For multivariate analyses, remaining missing values were imputed using variable-wise arithmetic means to obtain a complete matrix for ordination and clustering. Because variables differ in scale and units, all parameters were standardised using a z-score prior to distance calculations.

Multivariate structure was summarised using Principal Coordinates Analysis (PCoA) computed from Euclidean distances applied to the standardised matrix [32,33]. Hydro-chemical groups were identified using k-means clustering (k = 4) with 200 random initialisations [34], and a fixed random seed to ensure reproducibility. Cluster structure was visualised in PCoA space, including 95% confidence ellipses for groups containing three or more municipalities.

As an interpretative indicator relevant to brewing practice, the sulphate-to-chloride ratio (SO_4_^2−^/Cl^−^) was calculated post hoc using non-imputed concentrations and classified into chloride-forward (<0.5), balanced (0.5–1.5), and sulphate-forward (>1.5) categories following established brewing science guidelines [1,8,35,36]. A brewer-oriented typology was constructed by combining SO_4_^2−^/Cl^−^ categories with mineralisation bands defined by tertiles of the first PCoA axis. Spatial visualisation was performed by joining results to municipal boundaries and exporting outputs as ESRI Shapefile (UTF-8 .cpg encoding) to ensure compatibility with ArcGIS Pro and Quantum Geographic Information System (QGIS). All analyses were conducted in R software (Posit, MA, USA) using tidyverse- and sf-based workflows [37,38].

## 3. Results

### 3.1. Data Distribution

After data preprocessing and excluding municipalities that did not meet the predefined criteria, spatiotemporal cubes were constructed for each variable, indicating the number of municipalities incorporated for each variable. The resulting space-time cubes comprised 89 municipalities for SO_4_^2−^, 82 for Na^+^, 70 for Mg^2+^, 90 for Ca^2+^, and 83 for Cl^−^, while pH included a total of 243 municipalities.

**Figure 1:**
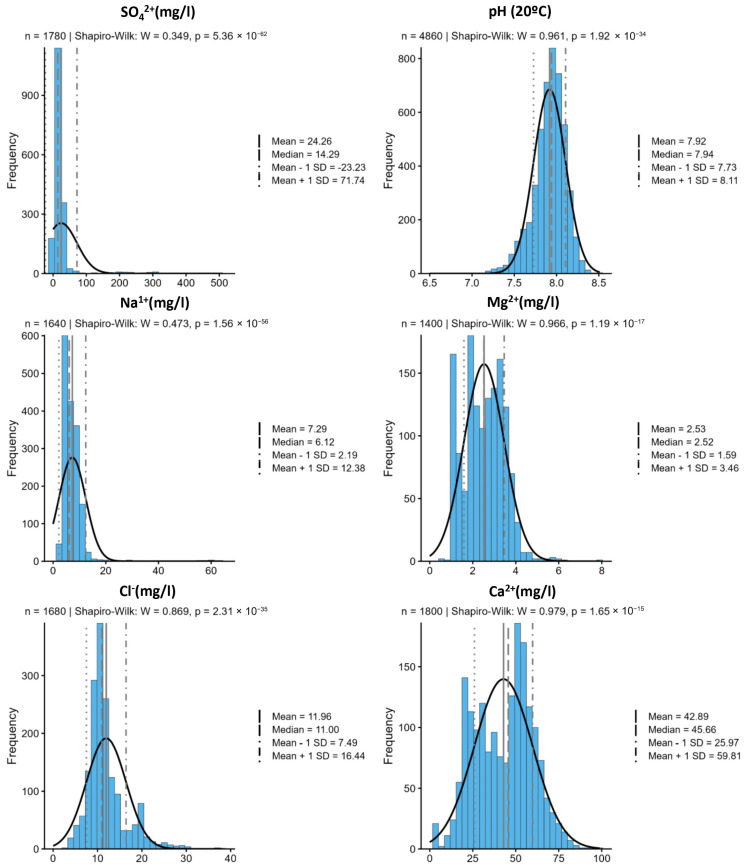
Frequency distributions of major ion concentrations and pH across municipal water supply points, illustrating variability and departures from normality.

The observed variability in major ion concentrations and pH across municipal water supply points highlights differences in water chemistry relevant to brewing applications (Figure 1). The maximum and minimum values of most of the studied variables and municipalities did not exceed the value ranges indicated in the brewing source water guidelines (Table 2), except for sulphate and sodium, which only occurred in two and one municipality, respectively and in less than the 0.06% of the records registered from 2019 to 2023.

### 3.2. Creation and Visualisation of the Space-Time Cubes

**Figure 2:**
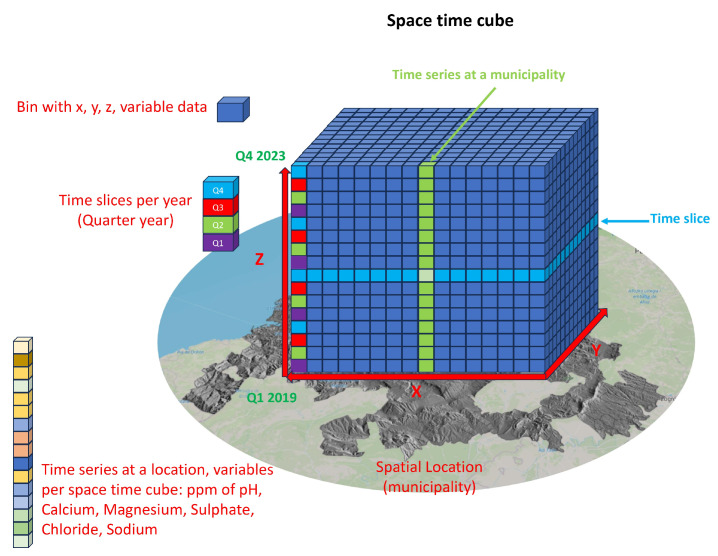
Structure of the six generated space-time cubes based on specified locations.

**Figure 3:**
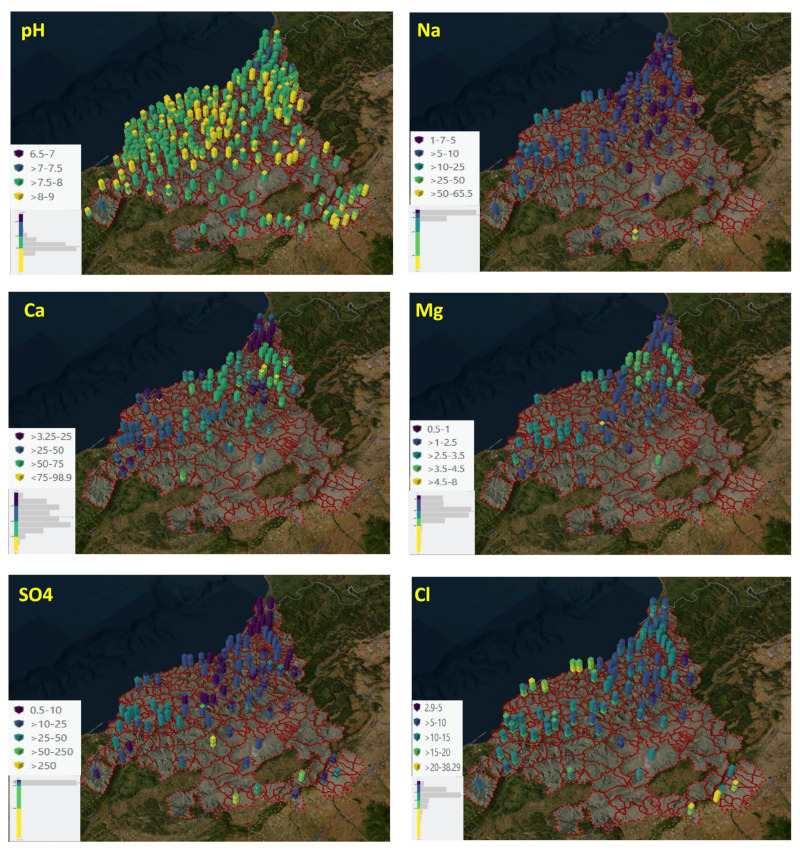
Space-time cubes of the studied variables in a 3D visualisation, illustrating the variable values (in ppm) at each municipality and quartile per year.

The space-time cube for each variable was created from the specified locations (Figure 2). The space-time cube model allowed for 2D and 3D visualisations, which facilitated the identification of spatiotemporal patterns. The 2D visualisation provided an overview of the overall trend throughout the study period, while the 3D showed each municipality’s historical state and changes (Figure 3).

### 3.3. Time-Series Clustering

**Figure 4:**
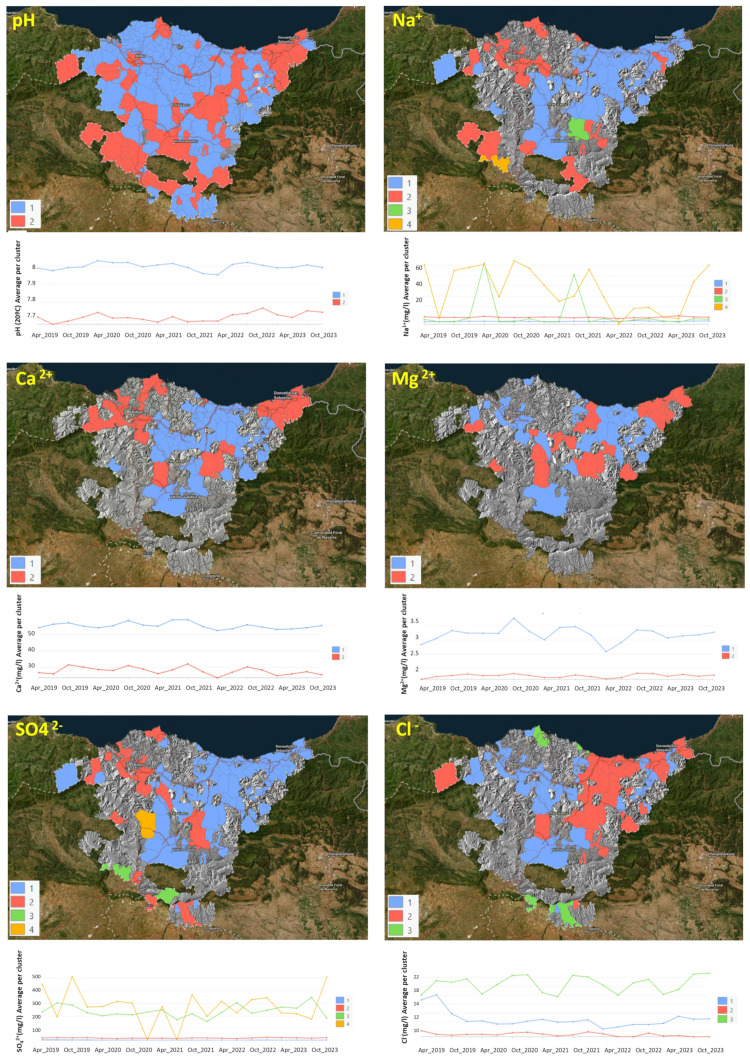
Time-series clustering maps showing similar values within the optimal number of categories, along with charts displaying the overall average time series, broken down by cluster using the mean.

Time-series clustering was applied to classify the municipalities in the space-time cubes using similar time-series characteristics in two to four clusters, showing similar smooth periodic patterns across time. The tool generated 2D maps showing each municipality in the cube symbolised by its cluster membership, and Enable Time-Series Pop-up parameters were used to create charts that showed the representative time series for each cluster and the time series for each location of the space-time cube (Figure 4, Table 3).

Time-series clustering revealed variable-specific spatiotemporal structures across municipalities. Sulphate and calcium exhibited stronger cluster differentiation, suggesting heterogeneous hydrochemical dynamics potentially associated with regional geological variability and water supply characteristics. In contrast, magnesium and chloride showed lower cluster separation, indicating more homogeneous temporal behaviour or reduced discriminative power linked to lower spatial coverage. Mann–Kendall analyses indicated that most temporal trends were not statistically significant, supporting the interpretation that medium-term hydrochemical conditions remained relatively stable during the study period. Among all variables, pH displayed the greatest temporal consistency and spatial coverage, resulting in the most robust clustering structure (Table 3).

Water quality assessment across the municipalities revealed several key findings regarding pH, magnesium, calcium, sodium, chloride, and sulphate levels. The pH ranged from 6.58 to 8.51. The municipalities were grouped into two main clusters based on pH levels: one with municipalities at a pH level closer to 8 and the other closer to 7.5.

Magnesium levels in the region ranged from 0.7 to 7.9 (within the water guideline range of 0–40 ppm), while calcium was between 3.5 and 98.8 (within the water guideline range of 50–150 ppm). The magnesium and calcium analyses also yielded two clusters, revealing similarities in their distributions. Although calcium did not show significant differences between the clusters, magnesium levels exhibited a significant upward trend in cluster 2, suggesting their potential regional variability.

Sodium concentrations varied significantly across the region, ranging from 1.7 to 64.5 ppm, exceeding the water quality guideline of 0–50 ppm. While most municipalities reported sodium levels below 25 mg/L, Lantarón showed elevated levels, indicating higher saline conditions in that area.

Chloride concentrations ranged from 2.9 to 38.3 ppm, remaining well below the upper water guideline of 100 ppm in the majority of the municipalities.

Sulphate concentrations varied widely, ranging from 0.5 to 510.8 ppm, with the water guideline set at a maximum of 250 ppm. Of note, two clusters with sulphate levels exceeding 100 ppm were identified in just three of the 89 municipalities, indicating specific areas with elevated concentrations may require further investigation.

### 3.4. The Emerging Hot Spot Analysis

**Figure 5:**
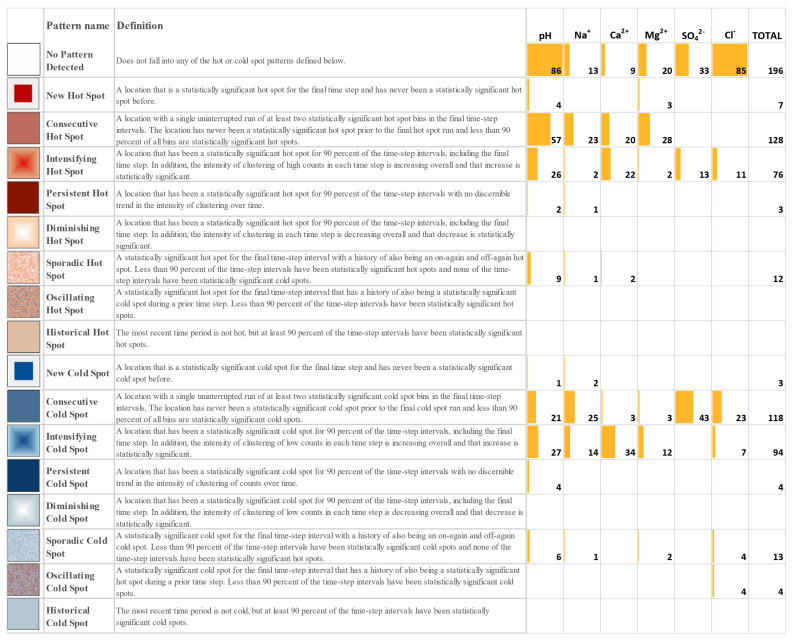
Frequency chart showing the spatial and temporal distribution patterns of each variable value and the total.

**Figure 6:**
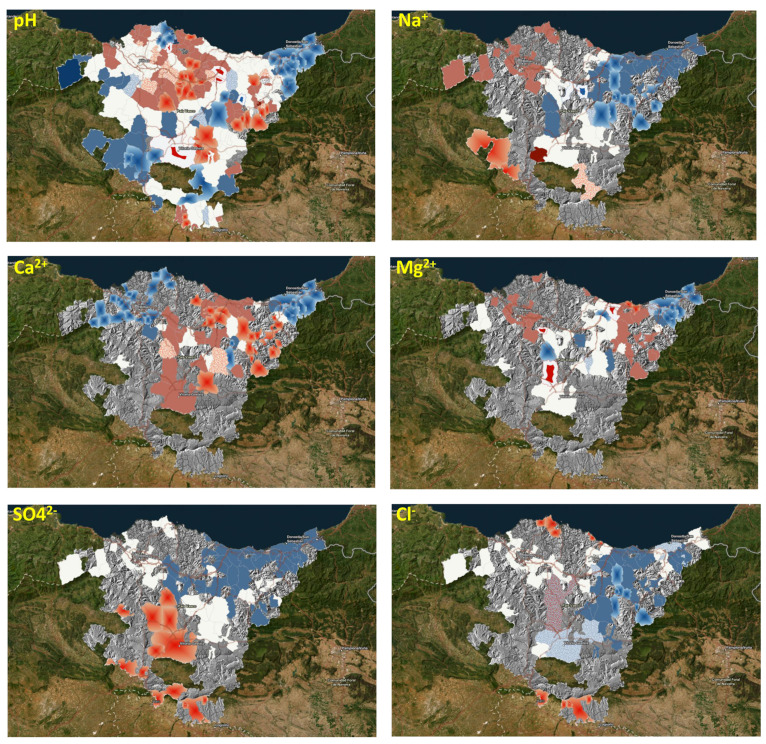
Spatiotemporal distribution of hot and cold values of each variable. Classification and description of hot and cold-spot patterns observed in the spatiotemporal analysis are shown in Figure 5.

In the context of Emerging Hotspot Analysis in ArcGIS Pro, particularly through the use of Space-Time Pattern Mining, the main objective was to identify regions where spatial patterns change over time, such as the emergence of hotspots or cold-spots. In this study, a total of eleven distinct spatiotemporal distribution patterns were identified, including stable distribution patterns of cold and hot spots (Figure 5 and Figure 6). Such analyses are essential for detecting evolving trends, spatial clusters, and anomalies. The absence of patterns typically suggests that the data lacks sufficient temporal and spatial coherence to reveal emerging hotspots or notable trends in the studied area. In this study, 35.21% of the data showed no discernible patterns.

Of the detected patterns, 31.83% represented hotspot patterns, where the values of certain variables were significantly higher than expected relative to surrounding areas and time periods. These patterns are critical for identifying emerging or expanding hotspots, which require further attention. In contrast, cold-spot patterns, indicating areas or time periods with significantly lower than expected values compared to their surroundings, were observed in 33.23% of cases. The patterns of consecutive hot and cold spots and intensified hot and cold spots were unevenly distributed across the study area.

For example, for pH variation, 35% of the municipalities exhibited no discernible patterns, indicating absence of high or low pH value clusters in these areas. However, 23% of the municipalities showed consecutive hot spots, with elevated pH levels across neighbouring regions and time periods, whereas 11% showed intensifying hot spots, signalling a growing concentration of high pH values. Conversely, 9% of municipalities displayed consecutive cold spots, indicating areas with consistently low pH values, and 11% exhibited intensifying cold spots, suggesting an increasing concentration of lower pH levels (Figure 5). The space-time hot spot analysis revealed persistent and intensifying pH clusters in specific areas, indicating sustained multi-year changes. These patterns are spatially coherent and correspond to zones likely influenced by shared treatment practices, such as alkalinity adjustment or source blending, which can affect pH levels. In contrast, emerging and fluctuating clusters appeared in areas potentially affected by climate-driven variability in raw water composition, including shifts in recharge and carbonate buffering conditions.

### 3.5. Forest-Based Forecast

The forest-based models were built for the entire space-time cube and were trained at all locations. Outliers in each time series were detected to identify the municipalities and times that deviate significantly from the patterns and trends of the remaining time series.

**Figure 7:**
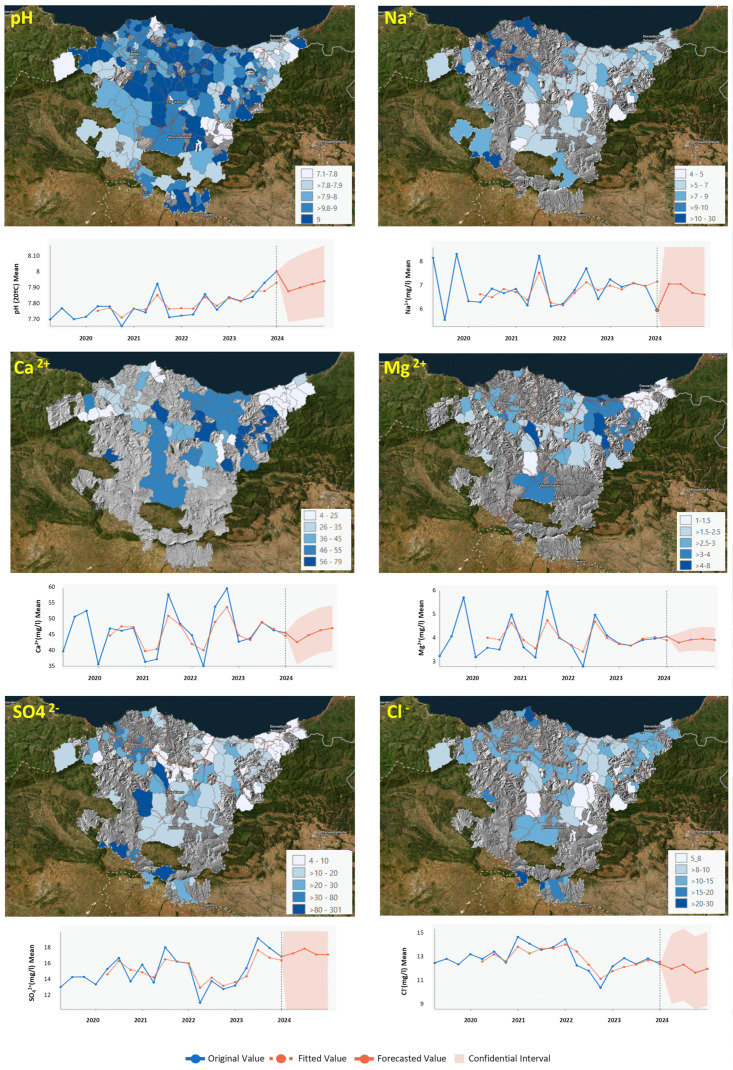
Maps of the final forecasted time step (2024) with pop-up charts from Vitoria municipality.

The primary output of the tool is a 2D feature class showing each location in the Input Space-Time Cube value symbolised by the final forecasted time step, with the forecasts for all other time-steps stored as fields for each studied variable (Figure 7).

Clicking any feature on the map using the Explore navigation tool in the ArcGIS Pro 3.2.2 software displays a line chart in the pop-up pane, showing the values of the space-time cube, including the fitted forest model, the forecasted values, and 90% confidence intervals for each forecast. In addition, it allows for the detection of outliers in the time series (Figure 7). Data from the municipality of Vitoria are presented as examples in Figure 7. The Forecast RMSE measures how well the forest model fits the raw time-series data, while the accuracy of the forecasts is evaluated using the Validation RMSE for each studied variable (Table 4). Since the Forecast RMSE uses more data and does not require extrapolation, it is typically smaller than the Validation RMSE (Table 4).

**Figure 8:**
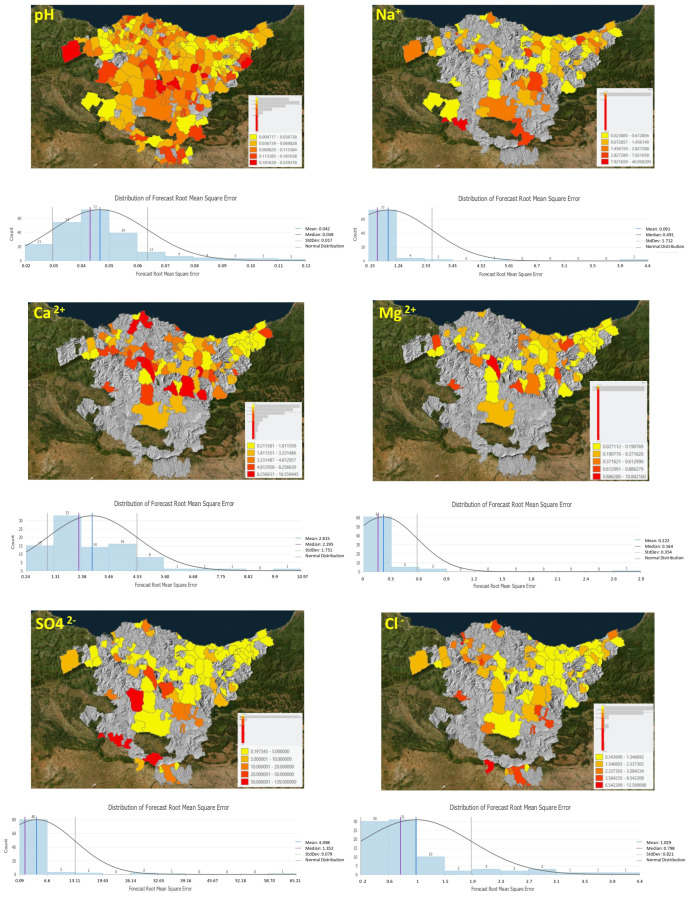
Spatial distribution of Validation RMSE values and frequency histograms of Validation RMSE and Forecast RMSE for the studied hydrochemical variables (pH, Ca^2+^, Mg^2+^, Na^+^, Cl^−^, and SO_4_^2−^). Maps represent the geographical variability of model validation errors among municipalities, whereas histograms summarise the distribution and dispersion of prediction errors across the study area. Lower RMSE values indicate higher model performance and temporal consistency, while higher values reflect increased spatial heterogeneity and forecasting uncertainty.

Model performance varied substantially among hydrochemical variables, reflecting differences in temporal stability, spatial heterogeneity, and data availability across municipalities (Figure 8, Table 4). Among all variables, pH showed the strongest predictive performance, with the lowest Forecast RMSE (mean = 0.04) and Validation RMSE (mean = 0.08), together with minimal dispersion of errors (SD = 0.02 and 0.05, respectively). The high number of analysed locations (218 municipalities) and space bins (4360) likely contributed to the robustness and stability of the forecasting model for this parameter.

Mg^2+^ also exhibited relatively low prediction errors, with mean Forecast and Validation RMSE values of 0.22 and 0.32, respectively, indicating relatively stable temporal dynamics despite lower spatial coverage. In contrast, Ca^2+^ and Cl^−^ presented intermediate forecasting performance. Calcium showed moderate prediction uncertainty (Forecast RMSE mean = 2.82; Validation RMSE mean = 5.48), suggesting higher spatial and temporal variability among municipalities, whereas chloride displayed more homogeneous error distributions with moderate overall RMSE values.

The largest forecasting uncertainty was observed for SO_4_^2−^. Although median Forecast RMSE values remained relatively low (1.35), very high maximum values were detected for both Forecast RMSE (63.42) and Validation RMSE (182.73), together with elevated standard deviations (9.08 and 27.7, respectively). These results indicate the presence of localised anomalies and strong hydrochemical heterogeneity in specific municipalities, potentially associated with geological factors or differences in water treatment processes.

Na^+^ also showed considerable variability in model performance. While median RMSE values remained relatively moderate, the large differences between median and maximum Validation RMSE values suggest positively skewed error distributions, indicating that most municipalities exhibited relatively low prediction errors whereas a limited number of locations presented substantially higher forecasting uncertainty.

Overall, variables with broader spatial coverage and greater temporal stability, particularly pH, produced more reliable forecasting performance, whereas variables characterised by lower data coverage and stronger local heterogeneity, especially SO_4_^2−^ and Na^+^, generated higher prediction uncertainty across the study area.

For the municipality of Vitoria, the pop-up charts in Figure 7 showed an increasing trend in pH, while Na^+^ remained relatively stable, with a slight decrease predicted for the period 2024-Q4. Ca^2+^ and Mg^2+^ exhibited similar temporal patterns, whereas Cl^−^ was expected to decline. The lowest Validation RMSE values were observed for pH and Mg^2+^, indicating the best model fit. Conversely, Ca^2+^ and SO_4_^2^ exhibited the highest Validation RMSE values, suggesting the poorest fit. These results highlight variability in forecast accuracy among water quality parameters and emphasise the importance of careful interpretation of model predictions. Despite these differences, the Forest-Based Forecast for Vitoria demonstrated generally low prediction errors across all variables. For pH, the forecast RMSE (0.035) and validation RMSE (0.102) reflect high predictive accuracy, and remain well below commonly accepted operational tolerances. Similarly, validation RMSE values for major ions were low: Na^+^ (1.296 mg L^−1^), Ca^2+^ (1.813 mg L^−1^), Mg^2+^ (0.443 mg L^−1^), SO_4_^2−^ (2.000 mg L^−1^), and Cl^−^ (1.347 mg L^−1^). These errors are small relative to concentration ranges typically relevant for brewing water composition, indicating that the forecast performance is adequate for practical assessment of water suitability and treatment requirements.

### 3.6. Hydrochemical Guidance for Beer Style Selection and Water Adjustment

**Figure 9:**
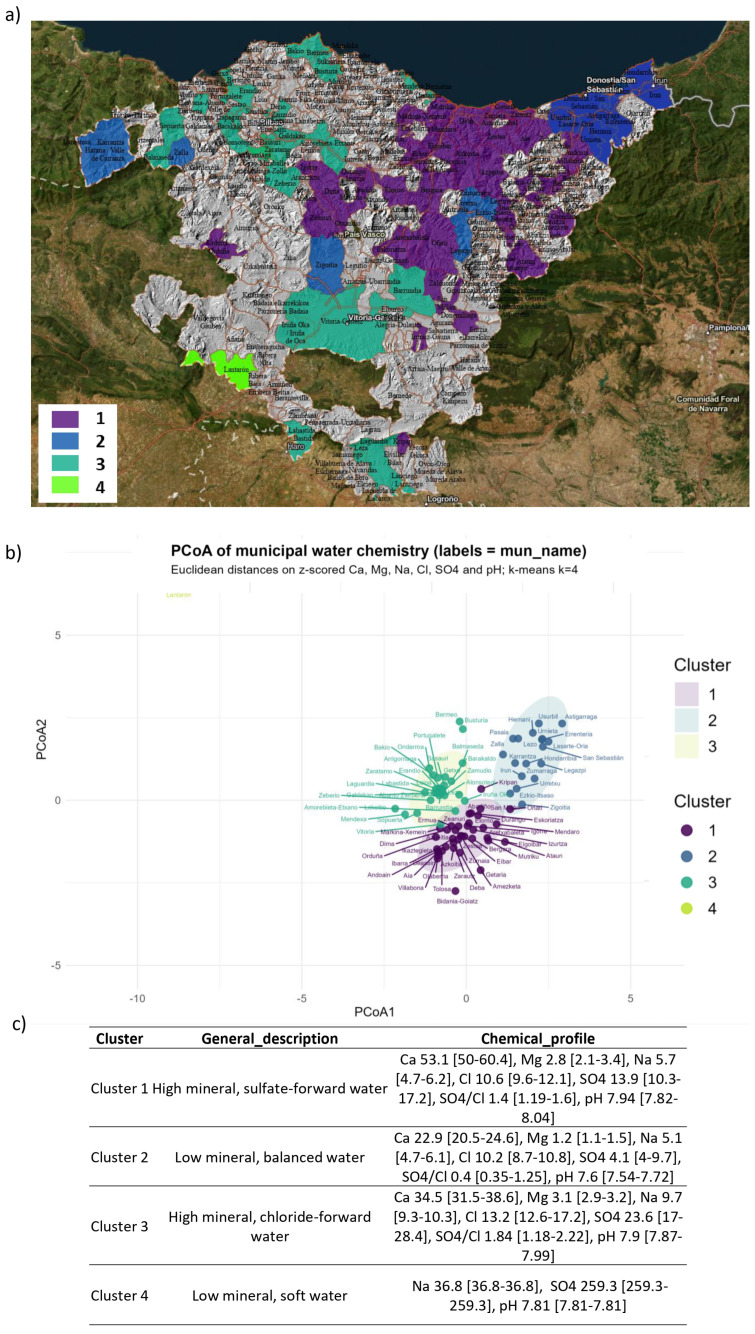
Multivariate analysis and spatial distribution of municipal water chemistry. (**a**) Spatial representation of municipalities coloured by to k-means cluster assignment (k = 4), illustrating geographic patterns of hydro-chemical similarity. (**b**) Principal Coordinates Analysis (PCoA) based on standardised concentrations of major ions (Ca^2+^, Mg^2+^, Na^+^, Cl^−^, SO_4_^2−^) and pH, showing hydro-chemical relationships among municipalities. (**c**) Summary of hydro-chemical cluster characteristics, including mean values and ranges of major ions (Ca^2+^, Mg^2+^, Na^+^, Cl^−^, SO_4_^2−^) and pH. Clustering was performed using multivariate chemical composition, followed by classification into brewing water types.

**Figure 10:**
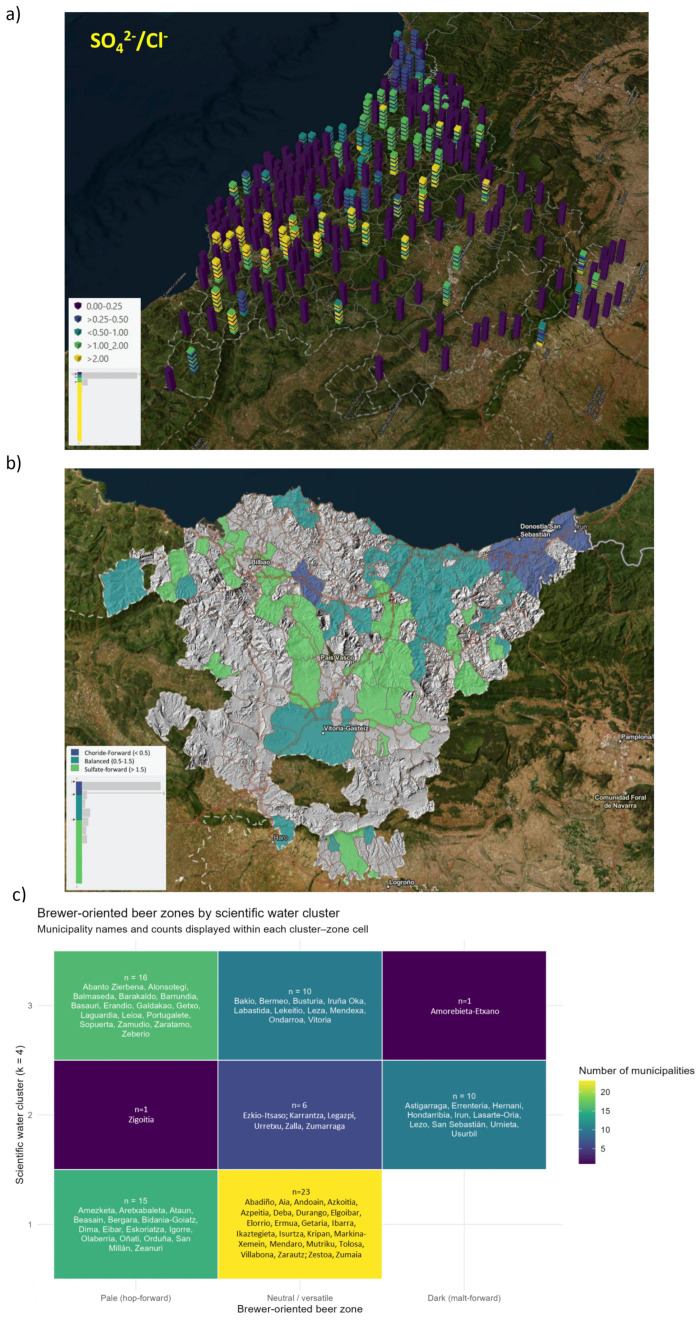
Integrated visualisation of sulphate-to-chloride balance and brewer-oriented water suitability. (**a**) Space-time cube visualisation of the sulphate-to-chloride ratio (SO_4_^2−^/Cl^−^) across municipalities, colour intensity indicates ratio magnitude, and vertical columns represent temporal aggregation. (**b**) Spatial classification of municipal brewing-water balance based on SO_4_^2−^/Cl^−^ ratio classes (**c**) Heatmap linking scientific water-chemistry clusters with brewer-oriented beer zones, enabling direct locality-level interpretation.

**Figure 11:**
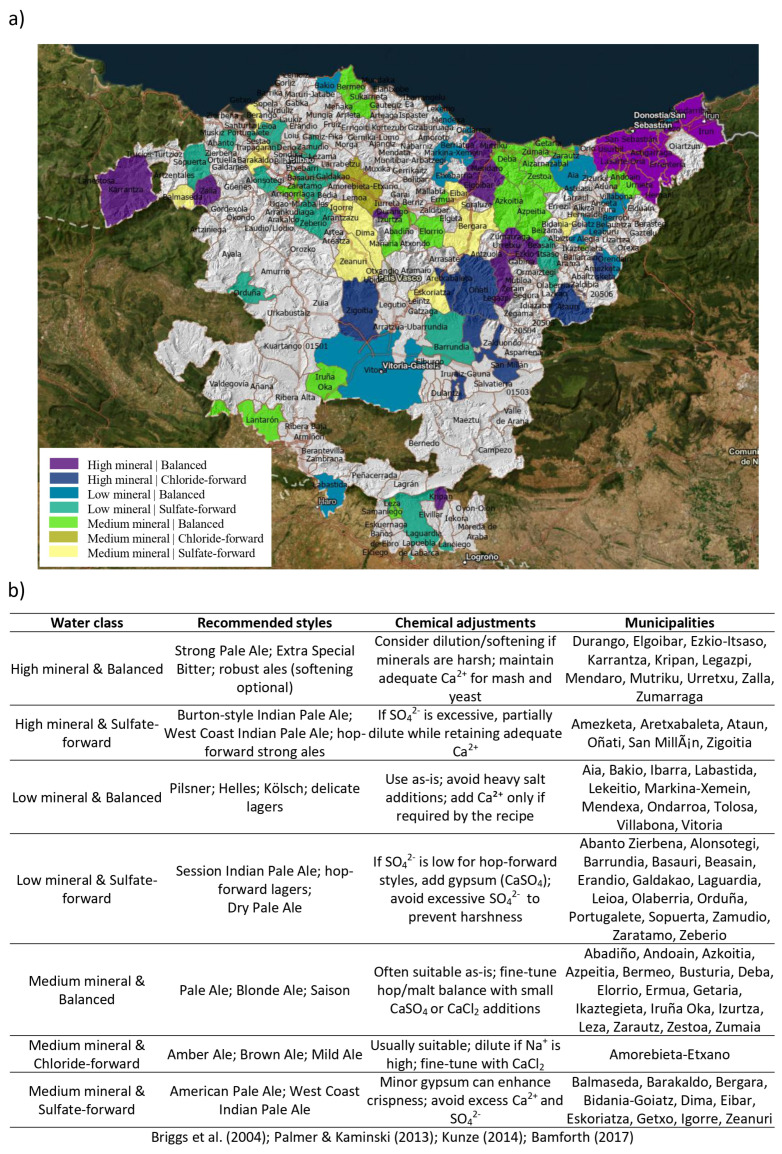
Water type classification for brewing applications (**a**) Brewer-oriented map integrating overall mineralisation level and sulphate-to-chloride (SO_4_^2−^/Cl^−^) balance. (**b**) Water classes, recommended beer styles, and required chemical adjustments per municipal groups [6,8,35,39].

The statistical analysis resulted in two complementary spatial representations: a scientific cluster map and a brewer-oriented cluster map. The scientific cluster map depicts classifications derived directly from multivariate analysis (PCoA and k-means), highlighting similarities in overall hydro-chemical composition based on major ions and pH (Figure 9). In contrast, the brewer-oriented cluster map translates these statistically defined clusters into applied water typologies by incorporating the sulphate-to-chloride (SO_4_^2−^/Cl^−^) ratio (Figure 10) and overall mineralisation levels, parameters known to influence sensory perception and brewing performance (Figure 11). While the scientific map emphasises objective chemical similarity, the brewer-oriented map provides an interpretative framework linking water chemistry to beer style suitability and potential water adjustment strategies. Together Figure 9, Figure 10 and Figure 11, link the statistical hydro-chemical structure with practical guidance for brewing water selection and adjustment. Additional spatial patterns reflect the influence of geological context on brewing suitability. Karstic hard waters, characterised by very high calcium concentrations and pronounced temporary hardness with moderate sulphate levels, are conducive to classic English ales and Pilsners when appropriate acidification is applied. Conversely, mountain-derived soft waters with uniformly low major ion concentrations, provide a neutral and highly adaptable base for delicate lager styles such as Pilsner, Helles, Kölsch, and other low-intensity beers, where minimal mineral interference is desired (Supplementary Figure S1).

## 4. Discussion

Regional water supply influences beer style development. This study mainly explores how different profiles of water from different locations impact the brewing process, with implications for process consistency and quality control. Six variables were selected for analysis based on their influence on the brewing process, as well as the quantity and quality of the variable database, according to the ArcGIS Pro toolsets. These toolsets enhance our comprehension of the space-time structure of the data and the cube aggregation process, while also supporting data-driven monitoring and decision-making. Additionally, they facilitate the visualisation of patterns and predictions of the variable values over time at specific locations of interest.

In the Basque Country, the current microbrewery locations and production styles are generally based on population centres (infrastructure, skills availability, and closeness to consumers) rather than on the availability of a suitable water resource [40]. Since microbreweries produce a wide style range, ascertaining the behaviour of key variables at spatiotemporal scale is crucial for ensuring consistent product quality and reducing process variability [5].

The hydrochemical composition of the studied waters resembles the soft, low-mineral brewing waters historically associated with Plzeň and some low-alkalinity brewing regions of southern Germany. The generally low concentrations of Ca^2+^, Mg^2+^, Na^+^, Cl^−^, and SO_4_^2−^ indicate a soft water profile comparable to classical Pilsen-type brewing water, traditionally linked to pale lagers and delicately flavored beers [35]. Although calcium levels were moderately higher than those of the historical Pilsen profile, the overall ionic composition remained much closer to Central European soft brewing waters than to highly mineralised sulphate-rich waters such as Burton upon Trent. Consequently, the studied waters appear particularly suitable for pale lagers, Helles, Kölsch, and balanced malt-forward beer styles, while their low mineralisation also provides high flexibility for brewing salt adjustments adapted to different brewing processes and beer styles.

The pH is a critical operational parameter affecting water quality. For effective disinfection, pH control is necessary during all phases of water treatment, making it a key factor in regulatory compliance and safe water supply. Optimal pH in different water sources varies according to the water composition and properties of the construction materials used in the distribution system. The pH of most drinking waters was within the range of 6.5–8.5, with varying mineral profiles, indicating general compliance with drinking water standards.

Calcium and magnesium affect hardness, whereas sodium, chloride, and sulphate are more important for flavour and process performance. Calcium is a crucial co-factor for the mash enzyme activity and contributes to protein coagulation. Magnesium accentuates flavour and sourness at lower concentrations but can become astringent and bitter at higher doses, potentially affecting product acceptability. It also affects mash pH, though its impact is less pronounced than that of calcium [8]. Water passes through several geological formations before reaching drinking water systems, thereby influencing its mineral content. Drinking water with high concentrations of calcium and magnesium, particularly magnesium, may reduce the risk of stroke [41]. In this study, the levels of both ions were below the concentrations specified by the water quality guideline [1], supporting their suitability for food processing applications.

Sodium accentuates flavour at lower doses but can produce undesirable sensory effects at higher concentrations and may also become toxic to yeasts, potentially compromising process performance. Most water supplies contain less than 20 mg of sodium per litre; however, in some countries, levels can exceed 250 mg/L. Sources of elevated sodium include saline intrusion, mineral deposits, seawater spray, sewage effluents, and salt used for road de-icing. In addition, water treatment chemicals such as sodium fluoride, sodium bicarbonate, and sodium hypochlorite can raise sodium levels to as high as 30 mg/L [42]. Most values of sodium in the study region were <25 mg/L, except in the municipality of Lantarón. This localised increase may be explained by geological factors, specifically the presence of a salt diapir [43,44]. From a quality control perspective, such local anomalies highlight the importance of site-specific monitoring and corrective strategies.

Chloride levels in unpolluted water are typically below 10 mg/L and occasionally below 1 mg/L [45]. Chloride in water can be considerably increased by treatment processes in which chlorine or chloride is used. Excessive chloride levels can increase corrosion in distribution systems [46], with implications for infrastructure integrity and water quality. In brewing, chloride contributes to product characteristics such as stability and mouthfeel [1], but its concentration must be controlled to avoid negative effects.

Sulphates are released into water from industrial waste and atmospheric precipitation; however, the highest concentrations are typically found in groundwater and are of natural origin. In Spain, the presence of sulphates in the water is due to the nature of the surrounding terrain. In the Basque Country, high sulphate concentrations were detected only in a limited number of municipalities, with most values remaining within guideline limits. However, exceedances observed in Lantarón and Zuia may affect brewing performance and flavour balance. In these cases, the water may be influenced by the evaporite diapirs of Añana and Badaya (Rio Baias), respectively. These localised deviations reinforce the need for continuous monitoring and, where necessary, water treatment interventions to maintain consistent product quality.

Differences in clustering performance and forecasting stability among variables were strongly influenced by spatial data coverage and temporal consistency. Variables with broader analytical coverage and relatively stable temporal behaviour, such as pH, produced more robust cluster structures, higher pseudo-F values, and lower forecasting uncertainty across municipalities. In contrast, variables with lower spatial coverage and greater local variability, particularly Mg^2+^ and Na^+^, showed weaker cluster separation and increased model variability, likely reflecting both reduced sample availability and higher sensitivity to localised hydrogeological conditions or changes in water supply sources. These results highlight the importance of data completeness and spatial representativeness when applying Space-Time Cube forecasting approaches to regional hydrochemical datasets.

Differences in Forecast RMSE distributions among variables likely reflect variations in temporal stability, spatial heterogeneity, and data coverage across municipalities. pH showed the most stable forecasting performance, whereas sulphate and sodium presented higher uncertainty, probably linked to localised geological and water-source variability. The predominance of right-skewed RMSE distributions indicates that forecasting errors were generally low for most municipalities but increased in a limited number of geographically heterogeneous locations. Prediction accuracy was generally higher for pH than for Mg^2+^ or Na^+^. This likely reflects the broader spatial coverage and lower temporal variability of pH measurements, whereas Mg^2+^ and Na^+^ showed more limited analytical coverage and greater local heterogeneity among municipalities, reducing model stability and increasing uncertainty.

In the study area, most of the surface is covered by carbonate-rich rocks, such as limestone and marl, while outcrops of base-poor materials, such as sandstones, schists, and granites, make up a smaller percentage [47,48].

The general homogeneity of water composition across the region could reflect, at least partially, broad geological and hydrogeological characteristics as well as water treatment practices. Although the present study was not specifically designed to perform a detailed geological or hydrogeological assessment, regional lithological and aquifer-related observations were considered as contextual elements to support the interpretation of the observed hydrochemical patterns. Areas with calcium-rich limestone formations typically yield “hard” water, whereas regions underlain by low-mineral-content rocks tend to produce “soft” water [49]. Geological structures, such as aquifers (e.g., sandstone or limestone aquifers), may also influence groundwater chemistry by affecting pH and mineral content according to the underlying rock composition [50]. Similar controls can affect surface waters and reservoirs, where shared catchment geology, hydrological regulation, residence times, and common water-supply infrastructures may contribute to comparable hydrochemical signatures across neighbouring municipalities. These combined geological, hydrological, and infrastructural factors may therefore help explain the spatial parallelism observed in the ion- and pH-based cluster maps generated in this study, although a dedicated quantitative geological–hydrochemical analysis would be required to statistically validate these relationships.

Water treatment processes, including filtration, softening, and mineral adjustments, are widely used to ensure consistent water composition, particularly when natural variability is significant. Technologies such as reverse osmosis effectively remove minerals from water, resulting in water with very low mineral content. This water can then be remineralised by adding specific minerals to achieve the desired composition [51]. In regions with multiple water sources, blending is used to create a consistent water profile with uniform mineral content, ensuring that variations in mineral concentrations do not impact the final product (e.g., beer or soft drinks) [52].

The comparison between the scientific and brewer-oriented cluster maps obtained in this study highlights the importance of interpretative frameworks when translating hydrochemical data into practical applications. While statistically derived clusters capture objective similarities, their industrial relevance improves when key indicators such as mineralisation and the sulphate-to-chloride balance are considered. In particular, the SO_4_^2−^/Cl^−^ ratio emerges as a key discriminator that does not dominate the multivariate structure but provides essential context for sensory and process-related interpretation. This dual representation demonstrates that waters with similar multivariate signatures may differ substantially in brewing relevance, underscoring the value of integrating post hoc chemical indicators with multivariate classification.

While the ArcGIS Pro Space-Time Cube framework demonstrated strong potential for integrating spatial and temporal dimensions in regional hydrochemical monitoring, several methodological limitations should be considered. The main limitation stems from the heterogeneous availability of hydrochemical data across municipalities, as not all variables were consistently analysed across locations and monitoring periods. Some parameters contained substantial missing data, limiting the development of fully robust multivariate regional models. To minimise bias associated with uneven data coverage, separate univariate space-time cubes were generated for each parameter, maximising spatial and temporal data retention but restricting the simultaneous analysis of interactions among hydrochemical variables. 

In addition, using five-year temporal quartiles, while effective for reducing short-term noise and identifying persistent structural patterns, may mask interannual variability and recent changes in water sources or treatment processes. The use of municipal mean values also represents a necessary spatial simplification that may not fully capture local-scale heterogeneity within water supply systems. Furthermore, although regional geological and hydrogeological observations were considered as contextual support for interpreting hydrochemical patterns, detailed lithological and aquifer-scale analyses at individual water supply points were beyond the scope of the present study. Consequently, future studies specifically designed with harmonised multivariate datasets, more homogeneous monitoring coverage, and high-resolution geological, lithological, and hydrogeological information at the water supply-point scale will be necessary to achieve more robust quantitative analyses of the environmental drivers controlling regional water chemistry. Nevertheless, this study represents a first methodological approximation of the application of the Space-Time Cube framework to municipality-scale brewing water assessment, demonstrating its considerable potential as an exploratory, visualisation, monitoring, and decision-support tool for long-term hydrochemical management in brewing-oriented applications.

Additional methodological limitations of the Space-Time Cube (STC) framework should be considered when interpreting the present results. Although the aggregation of data into five-year quartile intervals reduced short-term analytical noise and facilitated the identification of persistent spatial and temporal patterns, this approach may also mask interannual variability and delay the detection of recent changes in water sources, hydrochemical dynamics, or treatment processes. In addition, separate univariate cubes were generated for each hydrochemical parameter to minimise bias associated with uneven missing-data distribution among municipalities and variables. While this strategy improved data retention and analytical robustness, it limited the direct assessment of spatiotemporal interactions among variables and coupled hydrochemical processes. Consequently, the STC approach should be interpreted primarily as a medium- to long-term structural trend monitoring tool that complements, rather than replaces, conventional annual analyses and detailed site-specific hydrochemical investigations.

## 5. Conclusions

The multivariate classification of municipal water chemistry revealed coherent hydrochemical patterns that can be effectively translated into practical brewing applications by incorporating mineralisation levels and the sulphate-to-chloride balance. This framework provides a reproducible approach linking statistical water classification with applied process considerations, supporting water quality monitoring and process control strategies in brewing.

At the regional scale, Basque Country municipal waters are generally low in minerals, with key chemical parameters largely within guideline limits, indicating overall compliance with drinking water standards. Localized exceptions, such as elevated sulphate and sodium levels, reflect geological influences and highlight the importance of targeted monitoring and risk-based management approaches. The predominance of soft waters provides a favourable baseline for brewing applications, while allowing controlled mineral adjustments where needed.

This study represents a first methodological approximation of applying the ArcGIS Pro Space-Time Cube framework to municipality-scale brewing water assessment, demonstrating its potential as a regional tool for spatiotemporal hydro chemical monitoring and brewing-oriented decision support. Despite limitations associated with heterogeneous data availability, missing values, and the use of municipal-scale averages, the methodology successfully identified persistent spatial patterns and temporal trends in brewing-relevant water chemistry.

## Figures and Tables

**Table 1:** *Z*-score–based significance thresholds used in temporal and spatial analyses. The Trend column reports the direction and significance of temporal trends derived from the Mann–Kendall test, while the Remarks column provides the corresponding interpretation of *Z*-scores in the context of Getis–Ord Gi* clustering.

C	Z-Score	*p*-Value	Trend	Remarks
−3	<−2.58	99%	Decline with 99% CL *	Cold spot with 99% CL
−2	−2.58~−1.96	95%	Decline with 95% CL	Cold spot with 95% CL
−1	−1.96~−1.65	90%	Decline with 90% CL	Cold spot with 90% CL
0	−1.65~1.65	—	Non-significant trend	Non-statistically significant hot or cold spots
1	1.65~1.96	90%	Up with 90% CL	Hot spot with 90% CL
2	1.96~2.58	95%	Up with 95% CL	Hot spot with 95% CL
3	>2.58	99%	Up with 99% CL	Hot spot with 99% CL

* CL = Confidence Level.

**Table 2:** Descriptive statistics of the variables.

Parameter	Range of Values (ppm)	Brewing Source Water Guideline Range (ppm) ^1^	Mean	Median	SD ^2^
pH	6.6–8.5	5–9.5	7.92	7.94	0.19
Ca^2+^	3.5–98.8	50–150	42.89	45.66	16.92
Mg^2+^	0.5–7.9	0–40	2.52	2.52	0.93
SO_4_^2−^	0.5–510.8	0–250	24.26	14.33	47.50
Cl^−^	2.9–38.3	0–100	11.96	11.00	4.47
Na^+^	1.7–64.5	0–50	7.22	6.12	4.80

^1^ Palmer and Kaminski, (2013) [1]; Eumann, (2006) [39]. ^2^ SD: standard deviation.

**Table 3:** Summary of the ArcGIS Pro Space-Time Cube Time Series Clustering analysis for the studied hydrochemical variables (pH, Ca^2+^, Mg^2+^, Na^+^, Cl^−^, and SO_4_^2−^). The table includes the random seed used for cluster initialisation, the number of municipalities (locations) analysed, the total number of space-time bins evaluated, and the pseudo F-statistic summary for candidate cluster solutions. The optimal number of clusters was selected according to the highest pseudo F-statistic and trend statistics for the average time series, following the ArcGIS Pro Time Series Clustering procedure.

Variable	SO_4_^2−^					Mg^2+^					pH				
Random seed	5184					7078					9602				
Number of locations analysed	89					70					218				
Number of space time bins analysed	1780					1400					4360				
Pseudo F-Statistic Summary	Number of Clusters	Highest Pseudo F				Number of Clusters	Highest Pseudo F				Number of Clusters	Highest Pseudo F			
	2	185.432				2	127.273				2	190.404			
	3	296.785				3	105.601				3	181.382			
	4	420.123				4	88.080				4	155.443			
	5	381.447				5	74.030				5	133.131			
	6	342.908				6	68.237				6	113.641			
	7	310.552				7	65.245				7	102.288			
	8	287.116				8	61.299				8	94.607			
	9	261.774				9	54.216				9	86.025			
	10	240.331				10	53.776				10	80.881			
Optimal number of clusters is 2 based on the highest pseudo F-statistic and Trend Statistics for Average Time-Series	Cluster ID	Direction	Man Kendal Statistic	*p*-value	Number of Locations	Cluster ID	Direction	Man Kendal Statistic	*p*-value	Number of Locations	Cluster ID	Direction	Man Kendal Statistic	Number of Locations	*p*-value
	1	Not Significant	−1.12	0.23	62	1	Not Significant	0.0000	1	38	1	Not Significant	−0.7462	156	0.4555
	2	Not Significant	0.5516	0.5813	24	2	Not Significant	0.8111	0.4173	32	2	Increasing	21.089	62	0.0350
	3	Not Significant	0.3569	0.7212	2										
	4	Not Significant	−0.3246	0.7455	1										
Variable	Na^+^					Ca^2+^					Cl^−^				
Random seed	3546					6406					2119				
Number of locations analysed	82					90					84				
Number of space time bins analysed	1640					1800					1680				
Pseudo F-Statistic Summary	Number of Clusters	Highest Pseudo F				Number of Clusters	Highest Pseudo F				Number of Clusters	Highest Pseudo F			
	2	128.607				2	170.055				2	39.998			
	3	125.628				3	135.883				3	59.593			
	4	170.457				4	127.989				4	48.710			
	5	158.796				5	106.480				5	41.848			
	6	155.832				6	92.684				6	37.991			
	7	162.795				7	79.340				7	35.229			
	8	146.745				8	71.343				8	35.208			
	9	144.717				9	66.598				9	31.389			
	10	146.519				10	60.541				10	30.330			
Optimal number of clusters is 2 based on the highest pseudo F-statistic and Trend Statistics for Average Time-Series	Cluster ID	Direction	Man Kendal Statistic	*p*-value	Number of Locations	Cluster ID	Direction	Man Kendal Statistic	*p*-value	Number of Locations	Cluster ID	Direction	Man Kendal Statistic	*p*-value	Number of Locations
	1	Increasing	1.7195	0.0855	52	1	Not Significant	−13.302	0.1834	49	1	Not Significant	−10.707	0.2843	41
	2	Not Significant	−0.1622	0.8711	28	2	Not Significant	−15.249	0.1273	41	2	Decreasing	−21.738	0.0297	35
	3	Not Significant	−0.0332	0.9735	1						3	Not Significant	0.8111	0.4173	8
	4	Decreasing	−18.493	0.0644	1										

**Table 4:** Forest-based forecasting accuracy across locations for each variable.

Variable	Category	Min	Max	Mean	Median	SD.	N° of Locations	N° of Space Bins
pH	Forecast RMSE	0.02	0.14	0.04	0.04	0.02	218	4360
	Validation RMSE	0.01	0.46	0.08	0.07	0.05		
Na^+^	Forecast RMSE	0.15	11.21	0.09	0.5	1.71	82	1640
	Validation RMSE	0.16	28.78	1.8	0.92	3.64		
Ca^2+^	Forecast RMSE	0.24	8.92	2.815	2.29	1.75	90	1800
	Validation RMSE	0.61	15.93	5.48	4.47	3.6		
Mg^2+^	Forecast RMSE	0.02	0.72	0.22	0.16	0.35	70	1400
	Validation RMSE	0.03	1.87	0.32	0.24	0.29		
SO_4_^2−^	Forecast RMSE	0.09	63.42	4.09	1.35	9.08	89	1780
	Validation RMSE	0.09	182.73	9.9	2.21	27.7		
Cl^−^	Forecast RMSE	0.21	4.36	1.03	0.8	0.82	84	1680
	Validation RMSE	0.34	12.59	2.07	1.59	1.97		

RMSE. root mean square error; SD. standard deviation.

## Data Availability

The original contributions presented in this study are included in the article/Supplementary Materials. Further inquiries can be directed to the corresponding authors.

## Supplementary Materials

The following supporting information can be downloaded at: https://www.mdpi.com/article/10.3390/foods15112021/s1, Figure S1: Optimal values of variables in water to optimally achieve different beer profiles.
