# Supplementary figures and images for: Application of Space-Time Cube Analysis to Brewing Water Resources: A Complementary Decision-Support Tool for Breweries
Source: Foods. 2026 Jun 4;15(11):2021. doi: 10.3390/foods15112021 (PMC13256462; doi:10.3390/foods15112021)

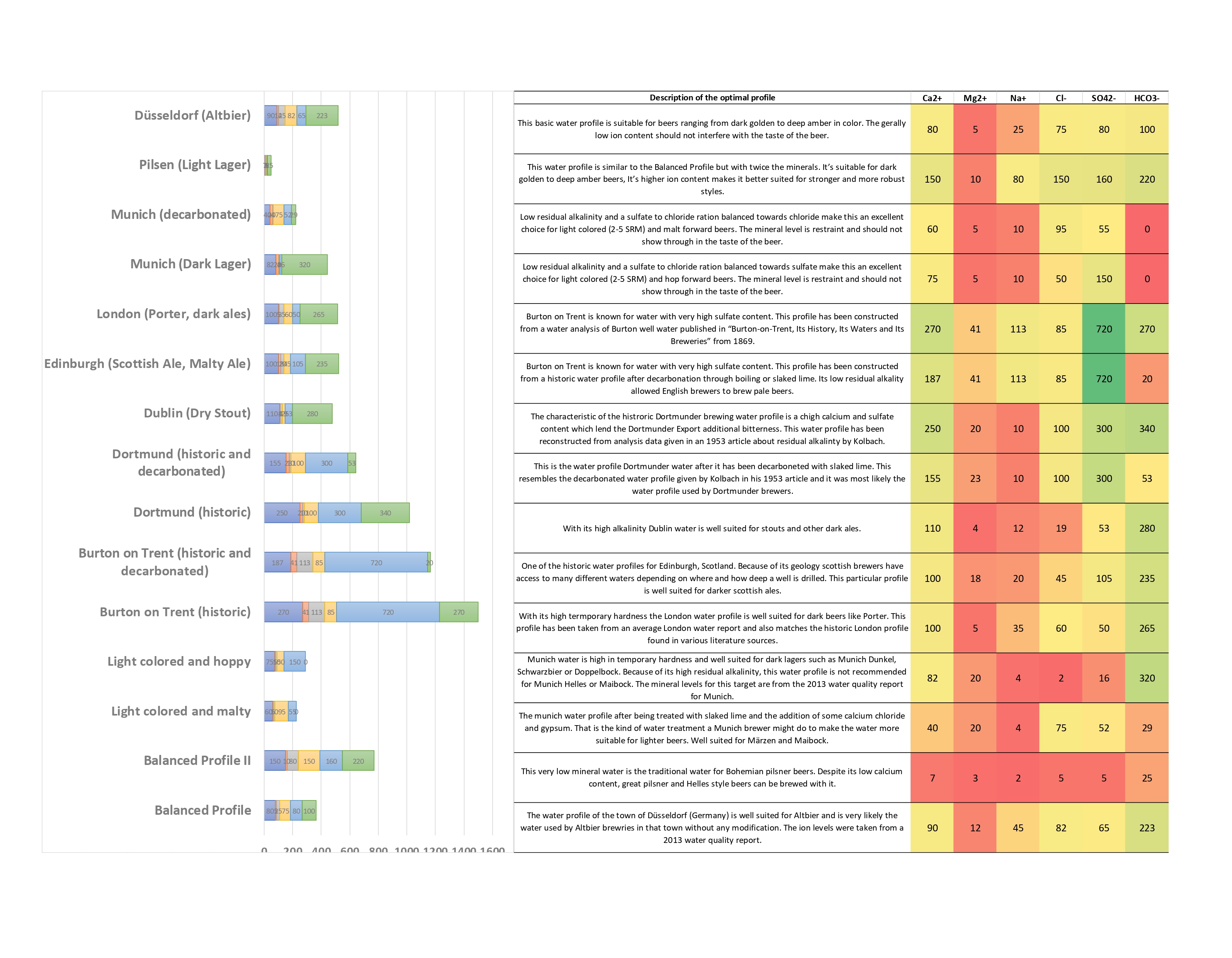

Supplement: Supplementary file 1 [file foods-15-02021-s001.zip › foods-4298586-supplementary.jpg]
